# Supplementary material for: Non-HLA Autoantibodies Against Angiotensin II Receptor 1 (AT1R) and Endothelin A Receptor (ETAR) in Pediatric Kidney Transplantation
Source: Int J Mol Sci. 2024 Nov 3;25(21):11817. doi: 10.3390/ijms252111817 (PMC11545982; doi:10.3390/ijms252111817)
Supplement: Supplementary file 1 [file ijms-25-11817-s001.zip › ijms-3262762-supplementary.pdf]

Supplementary materials

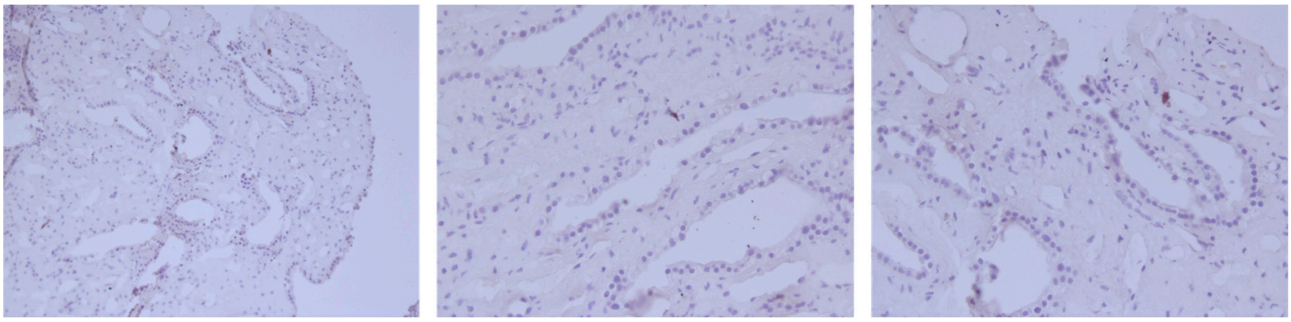

Figure S1: Immunohistochemistry on negative control

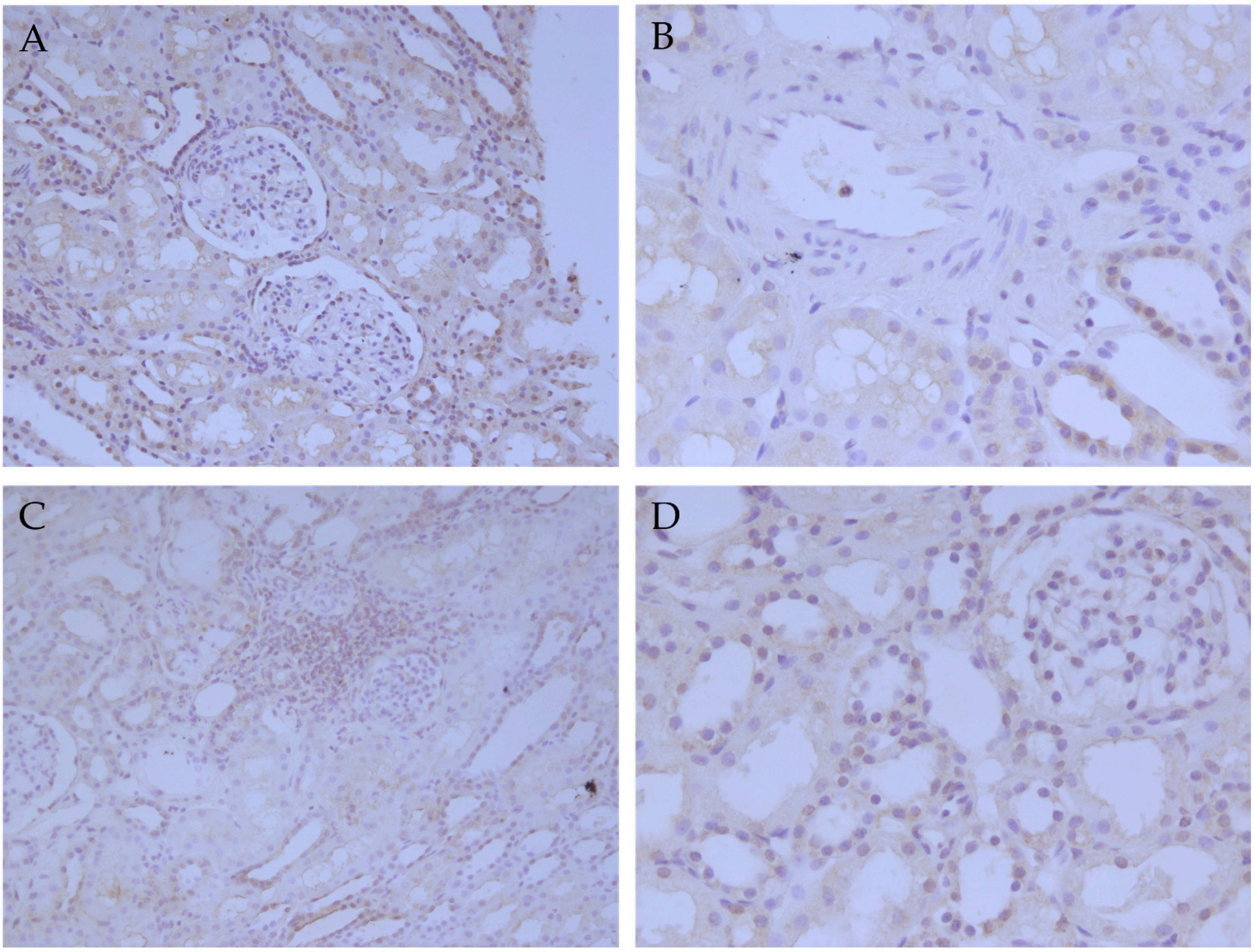

Figure S2: Histological localization of AT1R, Immunohistochemistry/ haematoxylin. A: glomerulus 20X B: artery 40X C: infiltrate 20X D: tubules 20X

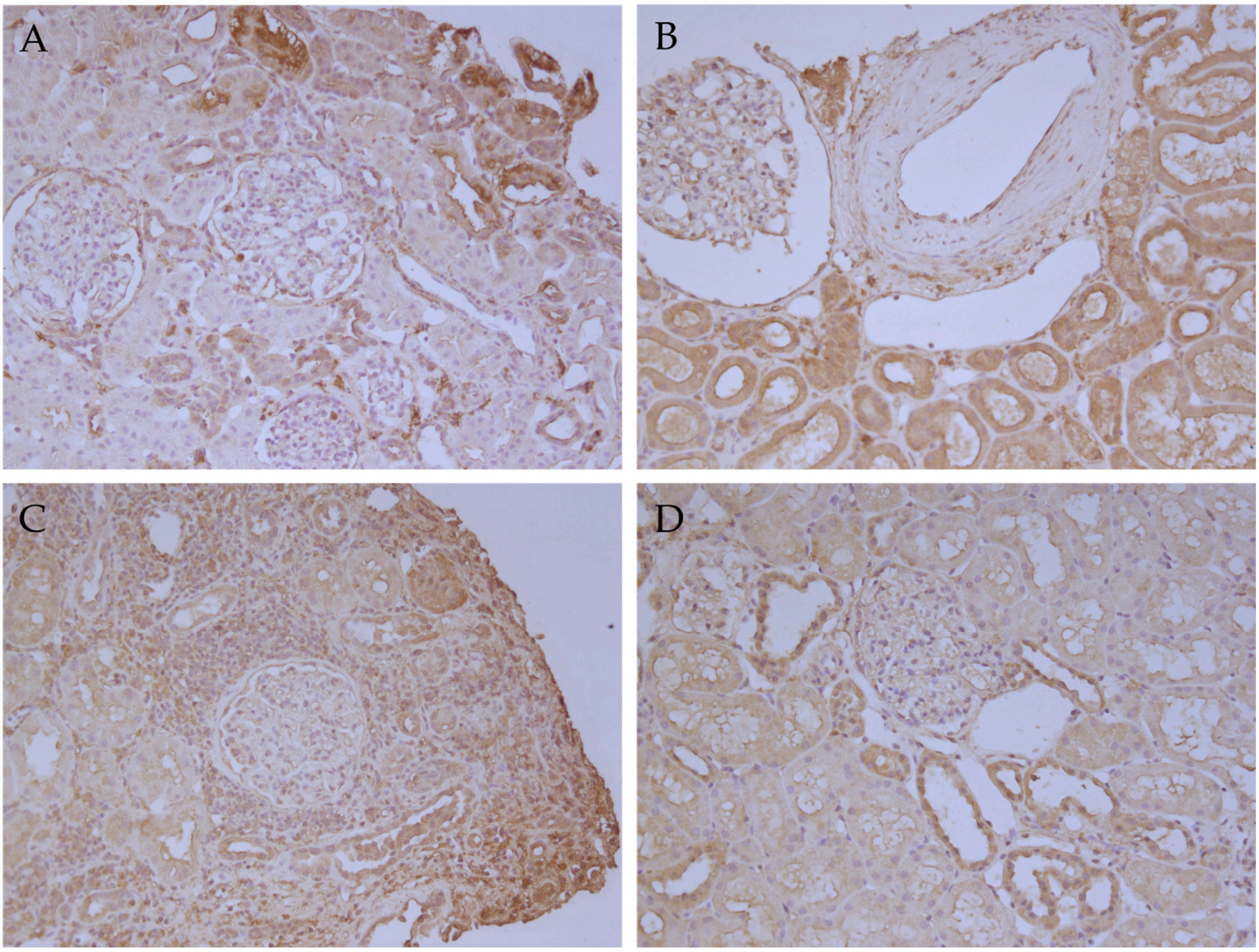

Figure S3: Histological localization of ETAR. Immunohistochemistry/ haematoxylin. A: glomerulus 20X B: artery 40X C: infiltrate 20X D: tubules 20X.

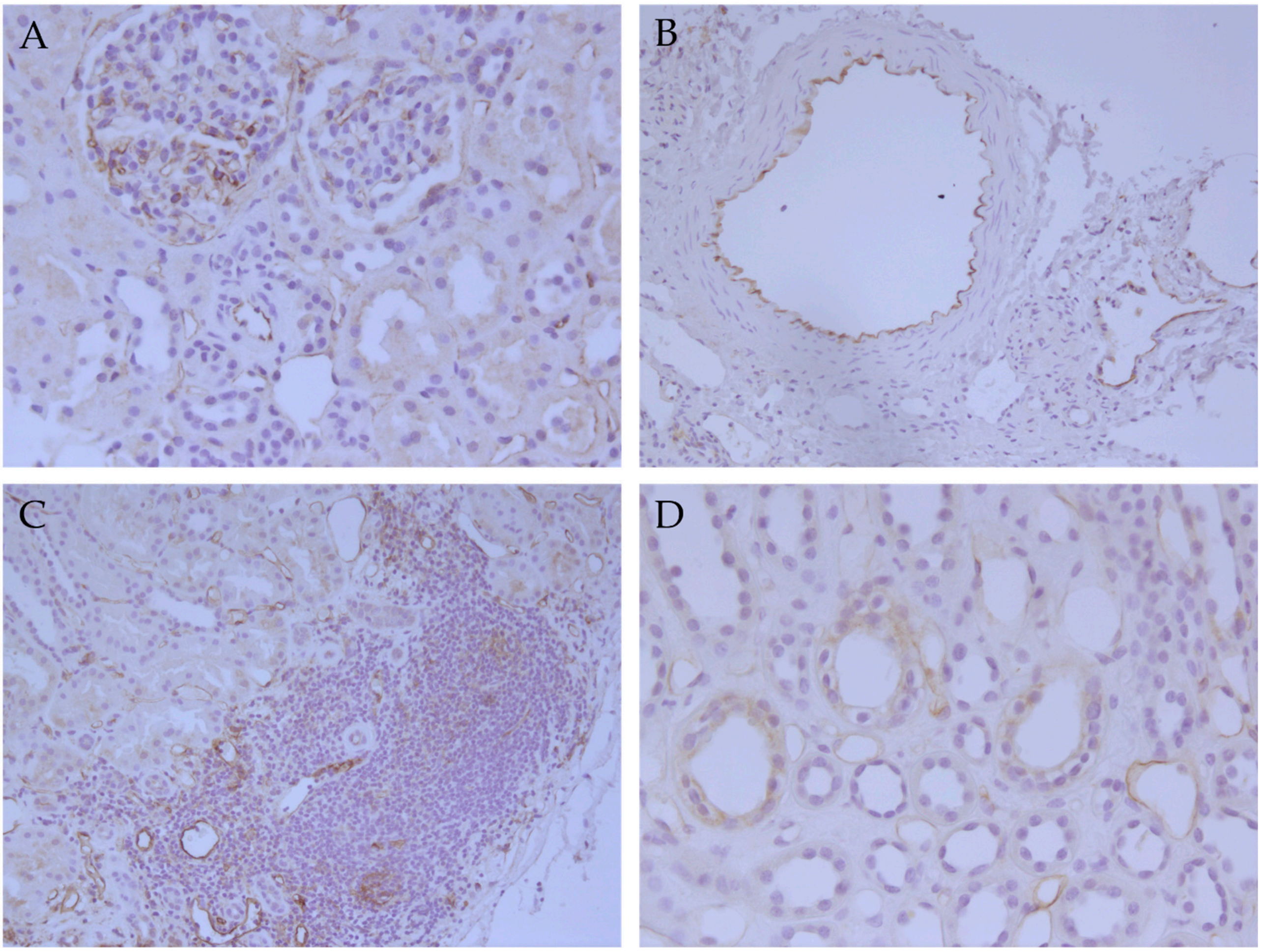

Figure S4: Histological localization of ICAM-1 Immunohistochemistry/haematoxylin. A: glomerulus 20X B: artery 40X C: infiltrate 20X D: tubules 40X

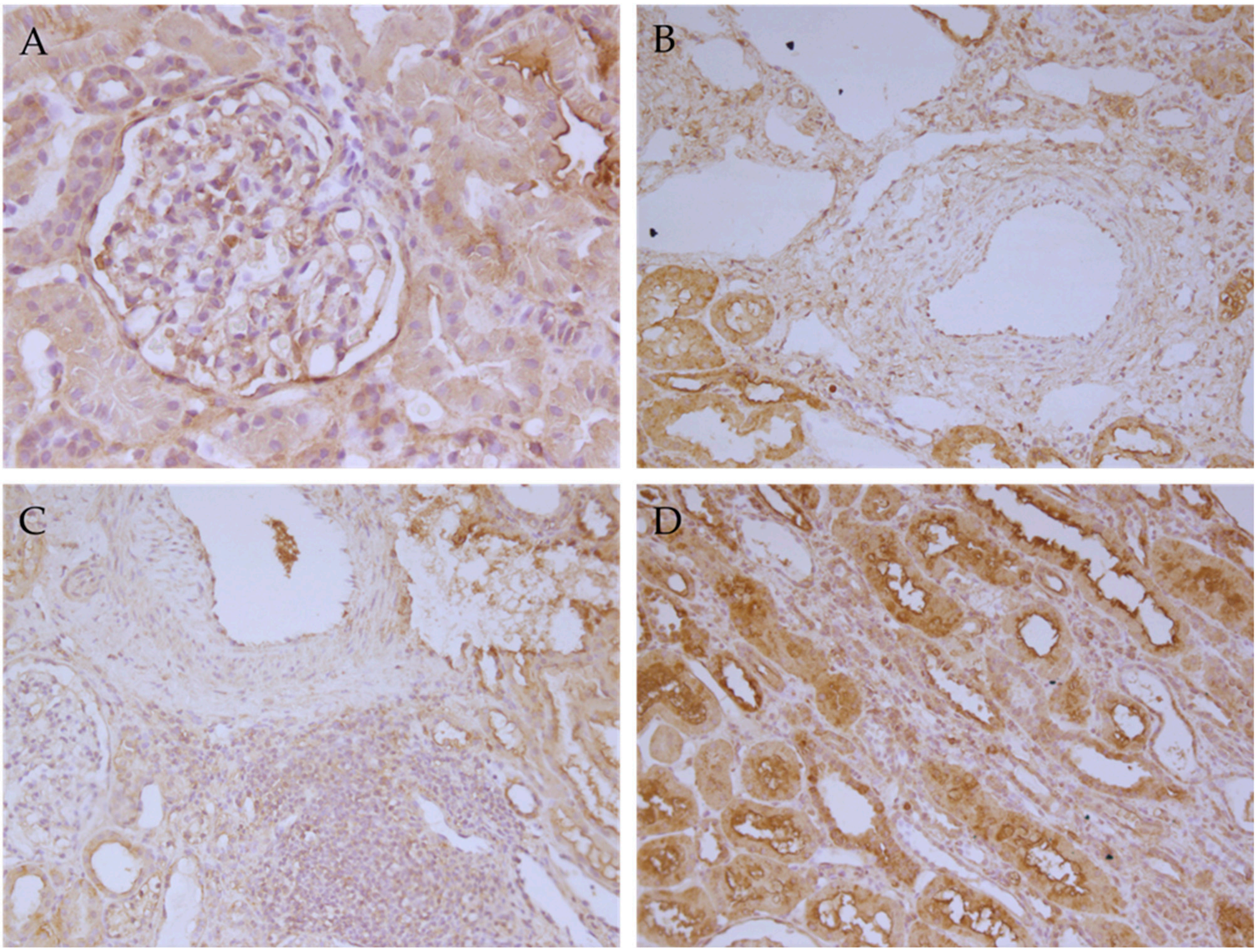

Figure S5: Histological localization of VCAM-1. Immunohistochemistry/ haematoxylin. A: glomerulus 40X B: artery 20X C: infiltrate 20X D: tubules 20X
